# CRISPR/Cas precision: do we need to worry about off-targeting in plants?

**DOI:** 10.1007/s00299-018-2355-9

**Published:** 2018-11-13

**Authors:** Florian Hahn, Vladimir Nekrasov

**Affiliations:** Plant Sciences Department, Rothamsted Research, Harpenden, AL5 2JQ UK

**Keywords:** CRISPR, Cas9, Plant, Off-target, Genome editing

## Abstract

The CRISPR/Cas technology has recently become the tool of choice for targeted genome modification in plants and beyond. Although CRSIPR/Cas offers a rapid and facile way of introducing changes at genomic loci of interest, its application is associated with off-targeting, i.e. introduction of unintended mutations at off-target sites within the genome, which has been reported frequently in the mammalian field. Here we summarise the current knowledge on the precision of CRISPR/Cas in plant systems and provide a summary of state-of the-art strategies for avoiding off-target mutations, as well as unintended on-target changes, in plants. These include using natural (e.g. Cas12a) or engineered (e.g. SpCas9-HF) CRISPR/Cas nucleases characterised by higher precision, as compared to the commonly used wild type SpCas9. In addition, we discuss the usage of CRISPR/Cas nucleases in the form of ribonucleoproteins (RNPs) as an option for reducing off-targeting in plants. Finally, we conclude that the most important factor for reducing CRISPR/Cas off-targeting remains careful selection of target sequences, for which we provide an overview of available online software tools and experimental guidance.

CRISPR/Cas (clustered regularly interspaced short palindromic repeats/CRISPR-associated protein) RNA-guided nucleases have recently become a valuable and versatile tool for genome editing applications in plants among many other organisms (Belhaj et al. 2015). Unlike other sequence-specific nucleases (SSNs), such as zinc finger nucleases (ZFNs) and TAL effector nucleases (TALENs), CRISPR/Cas can be easily engineered to recognise a DNA target of choice by manipulating the guide sequence within the guide RNA. Following the induction of a DNA break by CRISPR/Cas, the plant cell’s DNA repair mechanisms are exploited for generation of mutations. Despite of CRISPR/Cas becoming more and more popular with plant scientists, particularly as a reverse genetics tool, there is still a question of how precise this tool is and to what extend the issue of off-targeting, or any other unintended changes induced by CRISPR/Cas in the genome, is relevant for the plant field.

Similar to ZFNs and TALENs, CRISPR/Cas nucleases are not 100% precise and are able to cut DNA at off-target sites that share similarity with actual targets (Tycko et al. 2016). Within predicted off-targets, nucleotide mismatches affecting annealing of the so-called ‘seed region’ of the guide RNA (8–12 nt at the 3′ end of the 20 nt guide sequence in the case of Cas9) lower CRISPR/Cas activity significantly more than mismatches positioned towards the 5′ end of the 20 bp DNA target (Hsu et al. 2013). Since the protospacer adjacent motif (PAM; NGG in the case of SpCas9) is absolutely essential for CRISPR/Cas to recognise a DNA sequence, off-targets lacking the PAM motif are not targeted by CRISPR/Cas even if they are highly similar, or identical, to the actual 20 bp target (Hsu et al. 2013).

In the animal field, a lot of effort has been put into improving target specificity of CRISPR/Cas-mediated genome editing, including the use of double nickases (Ran et al. 2013), dCas9-FokI fusions (Guilinger et al. 2014; Tsai et al. 2014), Cas9 with shortened (17–18 nt) guide sequences within single guide RNAs (sgRNAs) (Fu et al. 2014) and a whole range of mutagenised ‘high fidelity’ Cas9 versions, such as SpCas9-HF (Kleinstiver et al. 2016; Slaymaker et al. 2016). In addition to the widely used *Streptococcus pyogenes* Cas9, or SpCas9, there are other natural Cas9 variants, such as *Staphylococcus aureus* Cas9 (SaCas9) and *Streptococcus thermophilus* Cas9 (StCas9). SaCas9 and StCas9 require longer PAM motifs than SpCas9 (NNGRRT and NNAGAAW, respectively) and have now been successfully applied in plants (Steinert et al. 2015; Kaya et al. 2016; Wolter et al. 2018). Due to the longer PAMs, SaCas9 and StCas9 are expected to have fewer predicted off-targets and, consequently, higher specificity (Kaya et al. 2016). Cas12a (former Cpf1; TTTV PAM) is another CRISPR/Cas nuclease, which is more precise than SpCas9 due to its intrinsic properties of DNA recognition and cutting (Strohkendl et al. 2018).

Apart from CRISPR/Cas nucleases characterised by higher precision, using CRISPR/Cas as a ribonucleoprotein (RNP), which is made of e.g. Cas9 protein in vitro assembled into a complex with an sgRNA, proved to be an alternative strategy to lower off-targeting in mammalian cells (Zuris et al. 2015). When delivered into the cell, RNP is less stable than DNA expressing CRISPR/Cas components and this decreases the time window, during which genomic DNA is exposed to CRISPR/Cas, resulting in lower off-targeting rates (Zuris et al. 2015).

In plants, the issue of CRISPR/Cas precision has not been addressed to the same degree as in mammals. This is mainly because off-target mutations can usually be relatively easily segregated away or removed by backcrossing in plants, which is a distinct advantage over mammalian (e.g. cell culture) systems. Additionally, in a polyploid species like wheat, CRISPR/Cas off-targeting can be used to one’s advantage if targeting all three homoeologues with the same sgRNA, while one of them differs from the other two by a single nucleotide polymorphism (Liang et al. 2017). Still, off-targeting can be highly problematic in reverse genetic studies and continues to be a concern also for plant scientists.

So far, a number of reports in plants clearly demonstrated that CRISPR/Cas is capable of introducing mutations into off-target sites, which share a significant similarity with targets (Lawrenson et al. 2015; Zhang et al. 2018; Tang et al. 2018). However, a comprehensive CRISPR/Cas off-target analysis on a genome-wide scale has been performed only in a few plant species, including rice, maize, tomato and Arabidopsis (Feng et al. 2014, 2018; Peterson et al. 2016; Nekrasov et al. 2017; Lee et al. 2018; Tang et al. 2018).

Tang et al. analysed specificity of both SpCas9 and Cas12a in rice using twelve SpCas9 sgRNAs targeting seven genes and three Cas12a CRISPR RNAs (crRNAs) targeting two genes, respectively (Tang et al. 2018). To estimate off-targeting rates, the authors performed whole genome sequencing (WGS) of control and CRISPR/Cas-mutagenised plants. The authors found that the majority of mutations they detected in CRISPR/Cas-expressing lines, or their progeny, were background mutations introduced during seed amplification or tissue culture (somaclonal variation). In addition to intended on-target mutations, only one out of the twelve Cas9 sgRNAs resulted in off-target mutations in a number of loci that shared significant similarity with the targeted locus and could be easily predicted using available online tools (Table 1). In the case of Cas12a, no off-target mutations were detected. Although only a limited number of Cas9 sgRNAs and Cas12a crRNAs were tested in this study, its results are consistent with other recent reports showing that CRISPR/Cas is highly precise when applied in plants (Nekrasov et al. 2013, 2017; Peterson et al. 2016; Feng et al. 2018; Lee et al. 2018).

**Table 1:** Online tools for CRISPR/Cas off-target prediction in plants

Online tool	Website	References
Cas-Offinder	http://www.rgenome.net/cas-offinder/	Bae et al. (2014)
Chop-Chop	http://chopchop.cbu.uib.no/index.php	Labun et al. (2016)
CRISPOR	http://crispor.tefor.net/	Haeussler et al. (2016)
E-CRISP	http://www.e-crisp.org/E-CRISP/	Heigwer et al. (2014)
CRISPR-P 2.0	http://crispr.hzau.edu.cn/CRISPR2/	Liu et al. (2017)
CCTop	https://crispr.cos.uni-heidelberg.de/	Stemmer et al. (2015)
Benchling	https://benchling.com/crispr	http://www.benchling.com
CRISPR-GE	http://skl.scau.edu.cn/	Xie et al. (2017)

Although occasionally off-targeting may be an issue when applying CRISPR/Cas in plants (Zhang et al. 2018), there is a number of strategies that one could use to minimise off-targeting. As already mentioned above, there are a few alternatives to SpCas9, which are expected to be more precise in plants either due to a longer PAM (e.g. SaCas9) or the actual molecular mechanism of DNA target recognition and cutting (e.g. Cas12a). A significant number of reports demonstrate that these CRISPR/Cas versions work well in species such as Arabidopsis, rice and maize (Lee et al. 2018; Wolter et al. 2018; Tang et al. 2018) and are therefore viable options for highly precise genome editing in plants. In addition, various ‘high fidelity’ mutant versions of SpCas9 (e.g. eSpCas9, SpCas9-HF) were applied in Arabidopsis (Zhang et al. 2018). Although these engineered SpCas9 versions are characterised by lower off-targeting rates in mammalian systems, their efficiency (i.e. on-target activity) in Arabidopsis seemed to be lower than efficiency of the wild type SpCas9 (Zhang et al. 2018). In addition, the RNP strategy has already been applied in several plants, such as wheat, maize and potato (Woo et al. 2015; Svitashev et al. 2016; Liang et al. 2017; Andersson et al. 2018). Nevertheless, many plant species are only stably transformable via Agrobacterium-mediated transformation, which is incompatible with the RNP strategy.

By far the most effective strategy to avoid CRISPR/Cas off-target activity is to make an informed decision about selecting the right CRISPR/Cas targets based on the genome sequence information from a particular plant species. Obviously, to perform a genome-wide off-target analysis, one needs the genome sequence. Preferably, the genome sequence should be annotated to prioritise off-targets that fall within coding regions. By now, there are various online tools available for one to select target sequences taking into account predicted on-target activity of CRISPR/Cas as well as putative off-targets (Table 1). As an example, Cas-OFFinder (Bae et al. 2014) has over ninety plant genomes in its database (with the possibility to request addition of further genomes) and allows one to choose a CRISPR/Cas nuclease for targeted mutagenesis (e.g. SpCas9, SaCa9 etc.) as well to specify the maximum number of mismatches in potential off-targets. Off-target sites with zero to two mismatches should be prioritised as they are more likely to be targeted unless the mismatches are located within the region pairing up with the sgRNA or crRNA seed (8–12 nt proximal to the PAM in the case of Cas9). Generally, the best target sites are the ones with a minimum number of predicted off-targets carrying few or no mismatches at all as compared to the target sequence.

When it comes to applying CRISPR/Cas for the purpose of genome editing, potential off-target effects are not the only issue of concern. Recently, Kosicki et al. have reported on unintended on-target changes, such as large chromosomal deletions, insertions and inversions, in mouse embryonic stem (ES) cells and human differentiated cells (Kosicki et al. 2018). Such chromosomal rearrangements are not always easy to detect unless long-range PCR or long-read next generation sequencing (NGS), such as PacBio, are used. Therefore, quite possibly, in many plant studies where targeted mutagenesis was performed using CRISPR/Cas, such unintended genomic changes might have remained undetected since the above-mentioned techniques were rarely used for genotyping CRISPR/Cas-induced mutations in plants. Usually, it is short-range PCR and/or a short-read NGS technology, such as Illumina, which are used for genotyping of mutagenised plant lines. Nevertheless, in plants, unintended changes, such as T-DNA or transformation vector fragment insertions into CRISPR/Cas cut sites within targeted genomic loci, have been reported (Xing et al. 2014; Andersson et al. 2017, 2018; Zhang et al. 2018). Zhang et al. initially failed to amplify across the *CPC* locus, which they targeted with CRISPR/Cas, in one of the mutagenised Arabidopsis lines (Zhang et al. 2018). The authors suspected a T-DNA insertion and, indeed, subsequently managed to detect it by PCR-amplification across T-DNA borders. In a different study, Andersson et al. observed that a high percentage of their potato CRISPR/Cas-mutagenised lines, which were produced by transforming protoplasts with a DNA vector, contained vector fragments inserted into CRISPR/Cas cut sites within the targeted *GBSS* gene (Andersson et al. 2017, 2018). Even after switching to the RNP strategy, where Cas9 was assembled into a complex with an in vitro transcribed sgRNA, the authors encountered a problem with fragments of the sgRNA encoding vector as well as random potato genomic DNA fragments being inserted into CRISPR/Cas cut sites despite of treating the in vitro transcribed sgRNA with DNAse I (Andersson et al. 2018). The authors eventually resolved the problem with unintended DNA inserts by switching to RNPs assembled using the Cas9 protein, and synthetically produced crRNA and trans-activating crRNA (tracrRNA) (Andersson et al. 2018).

In conclusion, there is a significant body of evidence suggesting that CRISPR/Cas is a very precise genome editing tool when applied in plants. In the majority of cases, off-targeting can be avoided by designing specific sgRNAs with the minimum number of predicted off-targets. Although a lot more research is required to figure out if unintended on-target changes, such as large chromosomal insertions, deletions or inversions, induced by CRISPR/Cas are a common phenomenon in plants, there is no doubt that CRISPR/Cas is a revolutionary technology that enables modifying plant genomes with unprecedented precision. Although CRISPR/Cas has its limitations, particularly when it comes to efficiency in many experimental setups in plants, in the future, it will certainly become a technology of choice for crop improvement and breeding, provided the regulatory and IP-related issues are overcome.
